# Unexpected involvement of a second rodent species makes impacts of introduced rats more difficult to detect

**DOI:** 10.1038/s41598-021-98956-z

**Published:** 2021-10-05

**Authors:** M. Lambert, S. Carlisle, I. Cain, A. Douse, L. Watt

**Affiliations:** 1 National Wildlife Management Centre, Animal and Plant Health Agency, Sand Hutton, York, YO41 1LZ UK; 2 Department for Environment, Food, and Rural Affairs, Foss House, 1-2 Peasholme Green, York, YO1 7PX UK; 3 Department of Life Sciences, Anglia Ruskin University, East Road, Cambridge, CB1 1PT UK; 4 NBC Environment, Federation House, 222 Queensferry Rd, Edinburgh, EH4 2BN UK; 5 NatureScot, Great Glen House, Leachkin Road, Inverness, IV3 8NW UK; 6 NatureScot, Rum Reserve Office, Isle of Rum, PH43 4RR UK

**Keywords:** Ecology, Zoology, Ecology

## Abstract

Rodent predators are implicated in declines of seabird populations, and removing introduced rats, often, but not always, results in the expected conservation gains. Here we investigated the relationship between small mammal (Norway rat, wood mouse and pygmy shrew) abundance and Manx shearwater breeding success on the island of Rum, Scotland, and tested whether localised rodenticide treatments (to control introduced Norway rats) increased Manx shearwater breeding success. We found that Manx shearwater breeding success was negatively correlated with late summer indices of abundance for rats and mice, but not shrews. On its own, rat activity was a poor predictor of Manx shearwater breeding success. Rat activity increased during the shearwater breeding season in untreated areas but was supressed in areas treated with rodenticides. Levels of mouse (and shrew) activity increased in areas treated with rodenticides (likely in response to lower levels of rat activity) and Manx shearwater breeding success was unchanged in treated areas (*p* < 0.1). The results suggest that, unexpectedly, negative effects from wood mice can substitute those of Norway rats and that both species contributed to negative impacts on Manx shearwaters. Impacts were intermittent however, and further research is needed to characterise rodent population trends and assess the long-term risks to this seabird colony. The results have implications for conservation practitioners planning rat control programmes on islands where multiple rodent species are present.

## Introduction

Predation, food supply and competition can regulate the size of animal populations, but determining the relative importance, or unimportance, of these different effects is notoriously difficult. Predation is at least partly responsible for driving multi-annual population cycles in snow shoe hares (*Lepus americanus*) and lemmings (e.g. *Dicrostonyx* spp.) for example, but even after decades of research, the exact role of predation, particularly in regulating lemming populations, is still not fully understood^1,2^. Predator and prey populations can be highly dynamic, and hence levels of predation may vary within and between prey populations over time creating difficulties in detecting and measuring the effects of predators. Predators are also subject to inter-specific and intra-specific effects including competition^3,4^ and interactions between multiple predator species may lead to unexpected (emergent) effects such as enhanced, substitutive or reduced (rather than simple additive or cumulative) impacts^5,6^.

Detecting (and mitigating) the effects of predators is of interest in conservation biology, and reversing the impacts of introduced predators on native wildlife is a major focus of conservation efforts worldwide. Introduced rats can have devastating effects on native seabirds, particularly on islands where mammalian predators were previously absent, and burrow-nesting seabirds may be particularly vulnerable^7,8^. Control or removal of introduced rats often results in tangible benefits to burrow-nesting seabirds, and control of ship rats (*Rattus rattus*) doubled the breeding success of Cory’s shearwaters (*Calonectris diomedea*) in Corsica for example^9^. However, elsewhere, this and other shearwater species persist in the presence of ship rats^10,11^. In another instance, removing introduced Norway rats (*Rattus norvegicus*) did not lead to the expected increase in numbers of sooty shearwaters (*Puffinus griseus*) or flesh-footed shearwaters (*Puffinus carneipes*) at a mixed colony in New Zealand^12^. The reasons why the impacts of rats apparently sometimes varies between seabird colonies are unclear, but it is possible that the relative importance of predation could vary geographically, with seabird colonies at the edge of a species' range unable to increase when rats are removed because bottom-up processes (such as habitat loss or food availability) are limiting^12,13^. Rat predation may not be the limiting factor for some seabird colonies, particularly where predation is intermittent, and introduced rats, like other predators, might also cause variable impacts on prey species over time which could be difficult to detect^14–16^.

In Britain, the (burrow-nesting) Manx shearwater is Amber-listed as a species of conservation concern and mainly breeds on the Welsh islands of Skomer, Skokholm and Middleholm, and the Scottish island of Rum^17,18^. Other islands off the British coast, including the Calf of Man and the Scottish island of Canna, historically supported large breeding colonies of Manx shearwaters, but these colonies have declined to near extinction, with introduced rats implicated in these declines^18^. Norway rats were removed from the island of Canna in 2005–2008 to conserve the remaining Manx shearwater colony^19^. Subsequent recovery of this Manx shearwater colony has been slower than expected however, with only one or two breeding pairs found in 2016, although populations of some other seabird species have responded positively to the removal of rats from the island^20^.

Norway Rats are also present on the island of Rum (adjacent to Canna) and have been linked to apparent declines of the Manx shearwater colony there^21^. Accordingly, removal of Norway rats from Rum has been identified as a priority action for Manx shearwater conservation in the UK^22^. Thompson (1987) however reported that Norway rats were not active predators of Manx shearwaters on the island, and that rats were prevented from becoming established in the shearwater colony by food shortages in late winter and the (upland) location of the colony within Rum. Controlling introduced rats or removing them from islands is a considerable undertaking that carries substantial costs^23,72^ and we wanted therefore to confirm whether introduced rats were negatively impacting on this globally important seabird colony.

Here we looked for associations between the breeding success of Manx shearwaters and the abundance of Norway rats and other small mammals over a four-year period on the island of Rum, and we tested whether localised use of rodenticides (which we expected would control Norway rats) resulted in increased shearwater breeding success. Our aim was to investigate whether rats negatively impact on the breeding success of Manx shearwaters on the island of Rum to inform the future conservation strategy for this seabird colony, but also to gain a better understanding of rodent-seabird interactions in upland habitats more generally.

## Methods

### Study sites

The study was carried out on the island of Rum (57°0’N, 06°20’W) which lies off the west coast of Scotland approximately 15 km south of the Isle of Skye and 25 km west of Mallaig. Rum is one of the inner-Hebridean Small Isles, a group that also includes the islands of Canna, Eigg and Muck. Rum (the largest island in the group) was designated as a National Nature Reserve (NNR) in 1957 and although some land has since been given over to the local community of approximately 30 residents, most of the island (approximately 108 km^2^) is still under the NNR designation and managed accordingly. Rum also has Special Protection Area (SPA), Special Area of Conservation (SAC) and Site of Special Scientific Interest (SSSI) designations which list the island’s protected areas and special features including important upland habitats and the globally important colony of Manx shearwaters^24^. The Manx shearwater breeding colony on Rum is reported to be the world’s largest, with breeding pairs preferentially occupying burrows and boulder crevices in well-drained volcanic soils along ridges and mountain tops above the 350 m contour^25^. A single egg is laid, usually in May, although the laying period extends into June, and incubation takes around 51–52 days^26–28^.

Like many islands, Rum has relatively few terrestrial mammal species; Clutton-Brock and Ball^29^ list these as red deer (*Cervus elaphus*), otter (*Lutra lutra*), brown rat (Norway rat), field mouse (wood mouse; *Apodemus sylvaticus*), pigmy shrew (*Sorex minutus*) and pipistrelle bat (*Pipistrellus pipistrellus*). Feral goats, a herd of ponies of uncertain origin (but of a similar breed to those found in the Scottish Western Isles) and a herd of Highland cattle are also present on the island and are managed to achieve appropriate grazing levels of plant communities in localised areas^24,29^. Red deer (deliberately reintroduced from mainland populations sometime after 1845) are managed to achieve a population density of 7–8 deer km^−2^ across the island although density is highest in the northern half of the island^24,29,30^. It is likely that both rodent species present were accidentally introduced; *Apodemus* probably from Scandinavia via Viking trade routes and the neighbouring island of Eigg^31,32^ while Norway rats were absent from Europe until the early eighteenth century^33^ and presumably reached Rum sometime after this. Norway rats are opportunistic feeders and survive independently of human habitation on Rum and many other islands globally^7^. On Rum, rats are most numerous around the coast, but they are also found inland, in woods and also open grassland where on Rum there is no competition from voles or house mice^34,35^.

**Figure 1:**
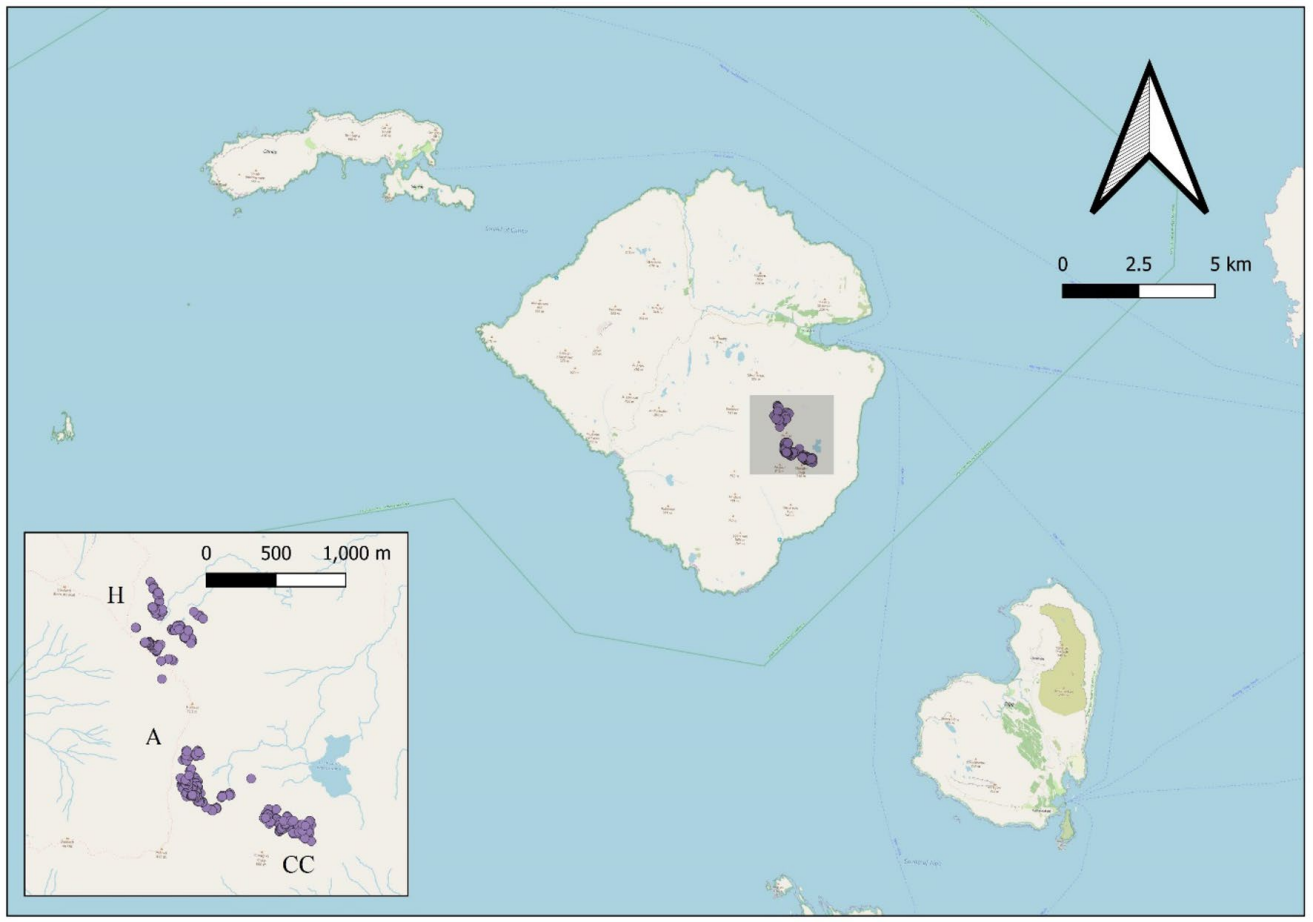
Location of the three study sites Askival (A), Hallival (H) and Clough’s Crag (CC) on the island of Rum, Scotland. Inset shows the location of the individual Manx shearwater burrows which were surveyed by endoscopy in early summer and again in late summer to generate an index of breeding success. Data were plotted using QGIS 3.16.3-Hannover (GNU General Public License). Map data copyright OpenStreetMap contributors, available under the Open Database Licence. Cartography licensed under the Creative Commons Attribution-ShareAlike 2.0 licence (CC BY-SA 2.0).

The study sites were located in an area of the Rum Cuillin in the southern part of the island (Fig. 1) in a varied upland landscape supporting a range of montane and sub-montane grassland, dwarf shrub heath and mire habitats^36^. The study sites each contained a group of (> 100) Manx shearwater burrows representing a spatially discrete compartment of the Manx shearwater breeding colony. Two sites (Askival and Hallival) were used in 2010, and a third site (Clough’s Crag) was added in 2011. Manx shearwater burrows were at least 250 m from the nearest burrow at neighbouring study sites. Within each study site a 30 m × 30 m grid was plotted using Hawth’s Tools in ArcMap 9.3.1 (Esri, California) and the grid points were uploaded onto handheld GPS units (Garmin, USA) for use in the field.

### Study design

We wanted to determine whether Manx shearwater breeding success was dependent on the abundance of Norway rats, and we tested whether Manx shearwater breeding success increased in response to rat control measures. Our study followed a similar design to that of Pascal, et al.^9^ where breeding success in sub-colonies of Cory’s shearwaters was compared before and after control of ship rats (*Rattus rattus*). Here we used rodenticides to control Norway rats and compared Manx shearwater breeding success in treated and untreated study sites. The study lasted for four years and we used a cross-over design (treatments were switched between sites after two years) to incorporate inter-site and inter-year variation.

### Manx shearwater breeding success

We inspected Manx shearwater burrows at each study site twice each year by endoscopy^37–40^. The initial (early summer) burrow checks were timed to follow on from the end of the egg-laying period, and hence started in mid-June each year, however the aim of these checks was to identify suitable study burrows and timing was not critical. We selectively chose Apparently Occupied Burrows (AOBs), i.e. those with signs of shearwater activity; digging, fresh droppings, nest material or one or more shearwater body feathers at the entrance^41^. Burrows were marked with a numbered tag and the location was recorded using a hand-held GPS unit. The selected burrows were checked again (by endoscopy) starting in mid to late August each year (late summer) when most eggs should have hatched, but before chicks had started to leave the nests^27^. The percentage of burrows that contained a live chick when re-checked in late summer was used as an index of breeding success^9^. Each year, ten burrows at the Askival study site were physically inspected (after endoscopy) as a comparison of the two methods. The initial (early summer) burrow checks took place in June and July each year but extended into mid-August in 2011 at the Clough’s Crag site. The final (late summer) burrow checks took place in the last two weeks of August each year but extended into the first week of September in 2011 at the Clough’s Crag site. The median number of days between checks for individual burrows was 55 (range 22–73).

### Monitoring rodent activity levels

We used indices of activity as a proxy for rodent abundance. The aims were to determine whether the rodenticide treatments were effective in controlling rats within the shearwater colonies and to investigate the relationship between rat abundance and Manx shearwater breeding success. In year one we trialled carbon-coated tracking plates^34,42^ concurrently with wooden chew sticks soaked in vegetable oil^15,43^. The rodent activity surveys were carried out by experienced field ecologists.

The tracking plates were deployed in 30 m × 10 m transects between 11 pairs of grid points at each study site. Each transect was subdivided into three 10 m × 10 m plots and four tracking plates were placed in each plot (resulting in a total of 132 tracking plates per study site per survey). We positioned the transects with the aim of sampling different elevations and the range of habitats represented within each study site, and within transects positioned tracking plates where they were most likely to detect rat activity; near watercourses, on obvious animal trackways and paths, at entrances to bait stations and near shearwater burrows.

Tracking plates also record footprints of other species and are recommended for evaluating the efficacy of rodenticide treatments against both rats and mice^44,45^. We therefore recorded whether the tracking plate had been marked by rats, mice or other animals, and also recorded a footprint score for each plate marked by rats based on the percentage the plate covered by rat footprints; 0% = 0; 1–25% = 1; 26–95% = 2; 96–100% = 3^42^. We carried out the tracking plate surveys in early summer, and again in late summer (within three weeks of Manx shearwater burrow checks) with tracking plates deployed for 2–3 nights each survey. Individual tracking plates were visually examined each day of the survey and replaced or repainted if marked.

For the chew stick surveys, one chew stick (soaked in vegetable oil overnight and secured in position using a metal tent peg) was placed at every accessible grid point (206 at Askival, 260 at Hallival) and checked in April, mid-May and late August. At the end of year one a decision was made on the most appropriate indexing method (chew sticks or tracking plates) based on success rate (number of rat detections). No signs of rat (or mouse) activity were recorded on chew sticks in year one and their use was therefore discontinued. The tracking plate surveys continued using the same method as in year one, and, in the absence of any obvious shift in the distribution of rat activity within each study site, the tracking plate transect locations remained fixed throughout the study.

### Control of Norway rats

We used rodenticides (containing coumatetralyl) dispensed from a 30 m × 30 m grid of bait stations (250–264 stations per study site depending on accessibility) with the aim of controlling Norway rats at one study site each year between 2010 and 2013. We used coumatetralyl (a ‘first-generation’ anticoagulant rodenticide; FGAR) because of the lower risk of (both primary and secondary) poisoning of non-target animals^46^. We expected that, due to a lack of previous exposure to rodenticides, Norway rats at the study sites would be susceptible to FGARs, and in a sample of 24 rats collected from the island shortly after the present study (unpublished data), we found none of the genetic mutations associated with resistance to anticoagulant rodenticides^47,48^. We considered wood mice to be a ‘non-target’ species^19^ and expected that use of a FGAR would reduce risks to wood mice^46^. Mice are generally less susceptible to anticoagulant rodenticides than rats and are most susceptible to second generation anticoagulants such as brodifacoum^49,50^. The Hallival study site was randomly assigned as the treated site in year one (2010) and was treated again in year two. Askival was assigned as the treated site in year three and was treated again in year four. Clough’s Crag was an untreated control throughout 2011 to 2013. Norway rat home range size is resource-dependent^51,52^ and hence we expected that rats would not routinely move between study sites during summer when food resources were more abundant (maintaining independence between study sites) and that increased movement of rats in response to declining food resources in autumn and winter (and seasonal mortality) would reduce carry-over effects between years.

Bait stations (each numbered, the locations logged in a GPS receiver) were 1 m lengths of 0.1 m diameter corrugated black plastic pipe secured in place with plastic ground anchors and heavy-duty nylon cord or weighed down with rocks. Following bait station deployment one (100 g) bait block (Romax rat CP, Barretine, UK) was placed in each bait station. The block formulation reduced risk of spillage and disturbance by wind, securing the blocks inside the bait stations reduced the risk of them being removed and discarded by rats. Bait station checks were carried out as required to maintain a surplus of bait and to check for and remove any dead rodents. Rodenticides were used in accordance with the label precautions by accredited pest control operatives (PCOs).

Decisions on the timing and the duration of the treatment were made iteratively and were based on observations from the rodent activity surveys in accordance with best practice guidance. In year one, the rodenticide treatment (at Hallival) was carried out mid-April to mid-May but after three weeks the rodenticide bait was removed (due to the low level of rat activity) and replaced with non-toxic monitoring blocks. There were some indications however (footprints on tracking plates, takes of non-toxic baits and suspected rat droppings in bait stations) that levels of rat activity increased towards the end of the shearwater breeding season at Hallival, and hence in subsequent years we extended the duration of the rodenticide treatment to cover the entire period between the first and second phases of shearwater burrow inspections. In year two the rodenticide treatment (again at Hallival) was started in late June (coinciding with the first phase of shearwater burrow inspections that year). In year three the rodenticide treatment (at Askival) was started in early June (three weeks before the shearwater burrow inspections commenced at that site) and in year four the rodenticide treatment (again at Askival) started in late April (eight weeks before the burrow inspections started at that site).

### Data analysis

We examined the effect of the rodenticide treatment on rat, mouse and shrew abundance by modelling the effect of site (Askival, Clough’s Crag, Hallival), year (2010, 2011, 2012, 2013), and season (early summer, late summer) on the number of tracking plates marked by rats, mice or shrews in Generalized Linear Mixed Models^53^ with a binomial distribution and logit link function (accounting for lost or unreadable plates in the binomial totals). Model terms were fitted sequentially and night of survey (1, 2, 3) was added as a random effect. The difference between early summer and late summer rodent activity gave an indication of the effect of the rodenticide treatment. For example, if, at treated sites, levels of rat activity significantly increased between early summer and late summer, we concluded that the rodenticide treatment had no effect on rats.

We calculated a General Index (GI) of activity for rats, mice and shrews for each survey period at each site by calculating the proportion of tracking plates marked per day, and then calculating the mean across survey days^54^. So, for example, if on night one at a study site, 22/132 tracking plates were marked by rats and 5/132 were marked by mice, and then on night two 20/130 tracking plates were marked by rats and 7/130 by mice, the index of activity for rats was 0.160 and the index of activity for mice was 0.046. For rats we also calculated an index of activity using the mean daily sum of the footprint scores for comparison with other studies^34,42,55^.

We used the general indices of activity to model the effect of rat, mouse and shrew activity in early summer and late summer on the index of Manx shearwater breeding success using General Linear Regression with site (Askival, Clough’s Crag, Hallival) and year (2010, 2011, 2012, 2013) added as fixed effects. Model terms were fitted individually, and a final model was selected by stepwise regression. We examined the effect of the rodenticide treatment on breeding success of individual Manx shearwater nests using a Generalized Linear Model (general model) with Bernoulli distribution and logarithmic link function^56^ with site and year added as fixed effects. Successful nests were those containing a live chick when re-examined in late summer. Data were analysed using GenStat 16th Edition (VSN International Ltd, Hemel Hempstead, Hertfordshire).

### Ethics statement

The study was approved by the Animal and Plant Health Agency’s Animal Welfare and Ethical Review Board (AWERB) and all methods were carried out in compliance with relevant guidelines and regulatory requirements.

## Results

### Rodent activity

In total we recorded 8,448 valid tracking plate scores (132 out of 8,580 possible observations were missing due to plates being lost or unreadable). In total, 154 (1.82%) tracking plates were marked by rats, 347 (4.11%) by mice, and 90 (1.07%) were marked by shrews (Table S1). Nine plates (0.1%) were marked by both rats and mice, two plates were marked by rats and shrews, two plates were marked by mice and shrews. No plates were marked by all three small mammal species.

Levels of rodent activity were generally lower in early summer than late summer. In early summer 57 out of 4,302 (1.3%) tracking plates were marked by rats, 114 (2.6%) by mice and 15 (0.3%) by shrews, whilst in late summer 97 out of 4,146 tracking plates (2.3%) were marked by rats, 233 (5.6%) by mice and 75 (1.8%) by shrews. The four-point index of rat activity (based on tracking plate scores) reached a maximum of 4.0 in early summer and 22.0 in late summer (Table 1). The mean (four-point) activity index across all sites and years was 1.9 (± 0.3 SE) in early summer and 3.5 (± 1.9 SE) in late summer.

**Table 1:** Rodent activity indices (AI) for three study sites (Askival (A), Clough’s Crag (CC) and Hallival (H)) on the island of Rum between 2010 and 2013. A rodenticide treatment (T) was carried out at one site each year (with the aim of controlling introduced Norway rats) while the remaining two were untreated (C).

Year	Site	T/C	General index of activity (GI)												Four-point index of activity			
			Rats				Mice				Shrews				Rats			
			Early summer		Late summer		Early summer		Late summer		Early summer		Late summer		Early summer		Late summer	
			AI	(SE)	AI	(SE)	AI	(SE)	AI	(SE)	AI	(SE)	AI	(SE)	AI	(SE)	AI	(SE)
2010	A	C	0.010	0.007	0.013	0.007	0.023	0.012	0.031	0.018	0.000	0.000	0.003	0.003	1.667	1.202	2.000	1.000
2011	A	C	0.008	0.005	0.004	0.003	0.033	0.008	0.038	0.011	0.003	0.003	0.046	0.012	1.000	0.577	0.500	0.500
2012	A	T	0.008	0.008	0.008	0.004	0.008	0.004	0.068	0.007	0.003	0.003	0.018	0.009	1.000	1.000	1.000	0.577
2013	A	T	0.010	0.010	0.013	0.003	0.020	0.011	0.053	0.008	0.000	0.000	0.010	0.007	1.667	1.667	2.000	0.000
2011	CC	C	0.013	0.005	0.000	0.000	0.049	0.014	0.039	0.022	0.008	0.004	0.063	0.023	2.000	0.577	0.000	0.000
2012	CC	C	0.025	0.013	0.033	0.015	0.013	0.007	0.010	0.007	0.010	0.003	0.000	0.000	4.000	2.000	5.667	2.906
2013	CC	C	0.020	0.010	0.138	0.007	0.013	0.005	0.018	0.007	0.005	0.005	0.015	0.012	2.667	1.333	22.000	0.577
2010	H	T	0.003	0.003	0.010	0.007	0.015	0.009	0.154	0.044	0.000	0.000	0.005	0.003	0.333	0.333	1.333	0.882
2011	H	T	0.010	0.005	0.000	0.000	0.051	0.007	0.053	0.013	0.000	0.000	0.013	0.009	1.333	0.667	0.000	0.000
2012	H	C	0.025	0.014	0.008	0.004	0.015	0.004	0.028	0.014	0.008	0.008	0.000	0.000	3.333	1.856	1.000	0.577
2013	H	C	0.013	0.003	0.023	0.008	0.053	0.023	0.114	0.017	0.003	0.003	0.038	0.015	1.667	0.333	3.333	1.202

There was a significant effect of rodenticide treatments however. Rodenticide treatments supressed levels of rat activity and were associated with an increase in activity levels of mice and shrews (Table 2).

**Table 2:** Estimates of the effect of rodenticide treatment on the number of tracking plates marked per night by rats, mice or shrews at three study sites on the island of Rum between 2010 and 2013.

	Untreated		Treated	
	Effect	*p*	Effect	*p*
Rats	0.6843	0.043	−0.0009	0.999
Mice	0.3799	0.087	1.3281	< 0.001
Shrews	1.5878	0.002	2.9110	0.002

A positive value indicates an increase in activity between early summer and late summer. Rodenticide treatment, site and year were included as fixed effects in the model (see Table S2).

### Manx shearwater breeding success

Of the 1511 AOBs checked in early summer 1199 (79.35%) were occupied (an adult egg or chick were seen by endoscopy), 1,349 were successfully re-checked in late summer and the index of breeding success was between 44.17 and 79.34%. (Table 3). For the 10 burrows that could be physically inspected, the endoscopy success rate was 92.86% (65 out of 70 endoscopy checks gave the same result as physical checks).

**Table 3:** Indices of Manx shearwater breeding success at three study sites on the Island of Rum between 2010 and 2013.

Site	Year	Sample size (AOBs)	Chicks	Index of breeding success
Askival				
	2010	118	73	61.86
	2011	128	81	63.28
	2012	130	85	65.38
	2013	121	69	57.02
Clough’s Crag				
	2011	115	73	63.48
	2012	121	96	79.34
	2013	115	54	46.96
Hallival				
	2010	120	53	44.17
	2011	139	83	59.71
	2012	120	86	71.67
	2013	122	56	45.90

Apparently Occupied Burrows (AOBs) were checked by endoscopy in early summer and then again in late summer.

**Figure 2:**
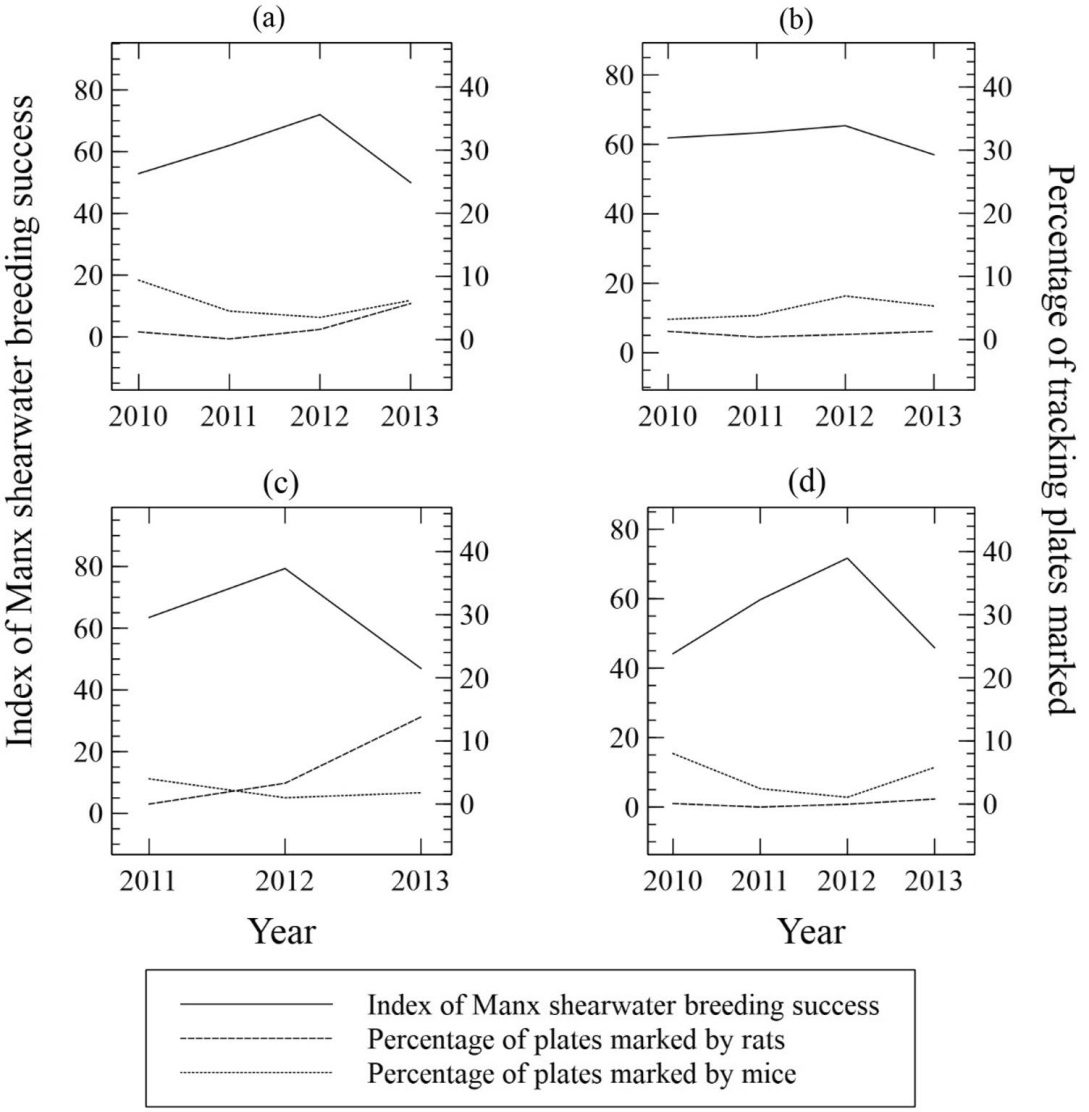
Relationship between percentage of tracking plates marked by Norway rats or wood mice and breeding success of Manx shearwaters (percentage of burrows containing a live  chick in late summer) at three study sites on the Island of Rum 2010–2013. Data are shown for the three study sites combined (**a**), and individually for each site Askival (**b**), Clough’s Crag (**c**) and Hallival (**d**).

Exploration of the data (Fig. 2) suggested a negative association between levels of rodent activity in late summer and Manx shearwater breeding success. General Linear Regression of the small mammal activity data against Manx shearwater breeding success, site and year indicated that Manx shearwater breeding success was negatively correlated with late (rather than early) summer levels of rat or mouse activity. The final model selected by stepwise regression (Table 4) (accounted for 92.9% of the variance in Manx shearwater breeding success and indicated that higher levels of rat (t = −3.42, *p* = 0.019) and mouse (t = −5.85, *p* = 0.002) activity in late summer were associated with lower levels of Manx shearwater breeding success. There was a significant year effect with Manx shearwater breeding success significantly higher in 2012. If late summer mouse activity was dropped from the final model, rat activity was a poor predictor of Manx shearwater breeding success (t = −0.28, *p* = 0.791), however the negative effect of mouse activity was still significant if rat activity was dropped from the final model (t = −2.87, *p* = 0.028). Late summer rat activity was not significantly associated with Manx shearwater breeding success if late summer mouse activity and year were dropped from the final model (t = −1.10, *p* = 0.299). At sites treated with rodenticides 290/510 (56.86%) nests were successful compared to 519/839 (61.86%) at untreated sites. The rodenticide treatments did not result in increased Manx shearwater breeding success (Table 5).

**Table 4:** Final model (chosen by stepwise regression) of the effect of late summer rat activity, mouse activity and year on Manx shearwater breeding success on the island of Rum 2010–2013.

Parameter	Estimate	SE	t	*p*
Constant	69.36	3.46	20.05	< .001
Rat activity	−113.5	33.1	−3.42	0.019
Mouse activity	−162.7	27.8	−5.85	0.002
Year 2011	0.03	3.08	0.01	0.992
Year 2012	10.37	3.07	3.38	0.020
Year 2013	−2.83	2.99	−0.95	0.388

Data were modelled by General Linear Regression.

**Table 5:** Estimates of parameters from a Generalized Linear Model of the effect of rodenticide treatments (‘Treatment’), study site and year on the success of 1349 Manx shearwater nests on the Island of Rum between 2010 and 2013.

Parameter	Estimate	SE	t	*p*	Antilog of estimate
Constant	−0.5462	0.0679	−8.04	< .001	0.5792
Treatment	−0.0858	0.0519	−1.65	0.098	0.9178
Site Clough’s Crag	−0.0119	0.0592	−0.20	0.841	0.9882
Site Hallival	−0.0839	0.0519	−1.62	0.106	0.9195
Year 2011	0.1254	0.0738	1.70	0.089	1.134
Year 2012	0.2821	0.0706	4.00	< .001	1.326
Year 2013	−0.0896	0.0818	−1.09	0.274	0.9143

## Discussion

Controlling or removing introduced rats is considered to be a valuable tool for seabird conservation on islands, and often, but not always, benefits seabirds and other native wildlife^12,23,72^. A better understanding of the reasons why some seabird colonies do not respond positively to the removal or control of rats is needed. Here, localised rodenticide treatments supressed activity levels of Norway rats during the Manx shearwater breeding season on the island of Rum, but did not result in increased Manx shearwater breeding success. We used a first-generation anticoagulant (coumatetralyl) with the intention of minimising risks to wood mice and pygmy shrews known to be present on the island, and activity levels of wood mice and shrews both increased in response to rodenticide use. Wood mice feed primarily on seeds, plant material and invertebrates in mainland contexts^57,58^ and we therefore regarded them as a non-target species^19^. We found however that activity levels of Norway rats and wood mice were both negatively correlated with Manx shearwater breeding success, and a model containing (late summer) indices of activity for both species provided the best predictor of Manx shearwater breeding success. Rat activity on its own was a poor predictor of Manx shearwater breeding success.

An association between activity levels of wood mice and Manx shearwater breeding success was unexpected, however wood mice have previously been reported as probable predators of Leach’s storm petrel (*Oceanodroma leucorhoa*) eggs^38^ and stable isotope analysis recently confirmed that wood mice consume seabird material, either through predation or scavenging^59^. It has also been reported that wood mice actively predate hibernating bats^60^. There was evidence that suppression of rat activity also increased levels of shrew activity but there was no evidence for an association between activity levels of shrews and Manx shearwater breeding success. We used activity indices from carbon-coated tracking plates as a proxy for rodent abundance. As a passive, indirect sampling technique, tracking plates are less likely to be influenced by seasonal or behavioural effects allowing for more robust comparisons of Norway rat activity between study sites and survey periods^61^. Other studies have reported a positive correlation between Norway rat activity indices from tracking plates and the number of Norway rats trapped^42^ or recorded by camera traps on farms in the UK^55,62^, and Carlisle^34^ found a positive correlation between capture-mark-recapture data and activity indices from tracking plates for Norway rats on the island of Rum. It should be noted however, that while the method we used to generate indices of abundance is recommended for assessing the impacts of rodenticide treatments on rats and mice^44,45^, further studies should confirm the relationship between wood mouse abundance and Manx shearwater breeding success using at least two indexing methods such as footprint tracking and capture-mark-recapture (C-M-R).

It has previously been reported that Norway rats suppress activity levels of wood mice on the island of Rum. Berry, et al.^35^ noted that damage by wood mice to young tree plantations declined after those areas were colonised by rats and Pankhurst^63^ reported that rats tried to access traps containing wood mice during live-trapping surveys, suggesting that rats likely predate wood mice on the island. Here controlling Norway rats led to increased levels of mouse activity, probably through competitor or mesopredator release^64,65^, and the replacement of the negative effects of Norway rats by wood mice likely explains why rodenticide treatments did not increase Manx shearwater breeding success.

It has previously been suggested that introduced rats should be removed from the island of Rum to conserve seabirds^22^. If wood mice also impact on Manx shearwater breeding success, and, given that they are a relatively recent (circa 1000 years ago) introduction to the island, the question arises of whether they should also be removed from Rum, or from other islands where they have been introduced, to restore native ecosystems. Where wood mice substitute the negative impacts of rats, removing Norway rats but not wood mice would likely not result in an increase in Manx shearwater breeding success. Removing mice is much more challenging than removing rats however; more attempts to remove house mice from islands fail^66^ and an attempt to remove wood mice from a Mediterranean island failed^67^. Public opposition to removal of wood mice is also likely to be greater than for rodents traditionally regarded as invasive pests such as Norway rats and house mice. It has previously been reported that the single-species removal of invasive vertebrates may be problematic, and, where possible, management initiatives should consider integrated management of invasive species^68^. However, this may not always be possible. In this case there would be a number of practical obstacles, not least that there are currently no rodenticide formulations approved for use against wood mice in the UK^69^. Alternatives to rodenticide use include trapping but this would likely be considered impractical on the scale that would be required here. On Rum, other introduced mammals have the potential to negatively impact on Manx shearwaters, including red deer^70^, although their populations are actively managed^24^. Interestingly however, historical ringing data suggest that the Manx shearwater colony on Rum is gradually increasing^71^, hence the current levels of impacts from rodents (or other introduced mammals) do not seem to be causing Manx shearwater declines, but they could be limiting potential increases, if other factors (such as food supply) are not limiting.

Here the distribution of both rats and mice varied spatially and temporally. Rodent activity levels were generally low in early summer compared to late summer. It is likely that upland rodent populations decline in late autumn as food resources become scarce and weather conditions deteriorate. The location of the Rum shearwater colony in resource-poor upland areas likely prevents continuous occupation by substantial numbers of rats. In most winters the (by then unoccupied) shearwater breeding grounds are covered by snow, and it is likely that rats retreat to lower altitudes below the snow line, possibly using the scattered woodland blocks as refugia^34^, and migrate back to higher altitudes during summer. However, it is also possible that small numbers of rats and mice persist at higher altitudes over winter and increase through reproduction during summer when weather conditions and access to food improve. Even in late summer however, the (four-point) index of rat activity based on footprint scores did not exceed 2.0 at any site (treated or untreated) during the first two years of the study. By contrast Quy et al.^42^ reported a mean activity index of 98.0 for Norway rats living in and around farm buildings. It is likely therefore that localised, intermittent rat control may be beneficial to the Manx shearwater colony on Rum, however the potential for the substitution of the effects of rats by wood mice, and the risk of exacerbating their effects, must be considered.

We conclude that the temporal variation in rat activity and the previously unknown involvement of wood mice provides a likely explanation for previous contradictory reports of negative effects of rats on this seabird colony. It is possible that similar temporally-mediated effects and undetected impacts from other species could provide explanations for some of the apparent geographical and temporal variation in impacts of introduced rats on seabird colonies elsewhere.

## Supplementary Information

Supplementary Information 1.

Supplementary Information 2.
