# Publisher Correction: Unexpected involvement of a second rodent species makes impacts of introduced rats more difficult to detect

**DOI:** 10.1038/s41598-021-00572-4

**Published:** 2021-10-28

**Authors:** M. Lambert, S. Carlisle, I. Cain, A. Douse, L. Watt

**Affiliations:** 1 National Wildlife Management Centre, Animal and Plant Health Agency, Sand Hutton, York, YO41 1LZ UK; 2 Department for Environment, Food, and Rural Affairs, Foss House, 1-2 Peasholme Green, York, YO1 7PX UK; 3 Department of Life Sciences, Anglia Ruskin University, East Road, Cambridge, CB1 1PT UK; 4 NBC Environment, Federation House, 222 Queensferry Rd, Edinburgh, EH4 2BN UK; 5 NatureScot, Great Glen House, Leachkin Road, Inverness, IV3 8NW UK; 6 NatureScot, Rum Reserve Office, Isle of Rum, PH43 4RR UK

Correction to: *Scientific Reports* https://doi.org/10.1038/s41598-021-98956-z, published online 05 October 2021

The original version of this Article contained errors in the layout of Table 1, where the heading “Four-point index of activity” was incorrectly shown as including the category “Shrews.” The incorrect and correct values appear below.

Incorrect:
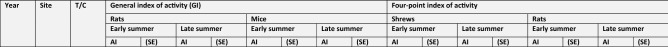


Correct:
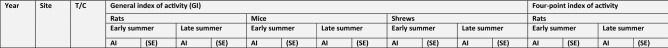


The original Article has been corrected.

