# Supplementary material for: Unexpected involvement of a second rodent species makes impacts of introduced rats more difficult to detect
Source: Sci Rep. 2021 Oct 5;11:19805. doi: 10.1038/s41598-021-98956-z (PMC8492617; doi:10.1038/s41598-021-98956-z)
Supplement: Supplementary file 1 — Supplementary Information 1. [file 41598_2021_98956_MOESM1_ESM.docx]

Table S1. Tracking plates marked by rats, mice or shrews at three study sites on the Island of Rum between 2010 and 2013.

|  |  |  | Early summer | | | | |  | Late summer | | | | |
| --- | --- | --- | --- | --- | --- | --- | --- | --- | --- | --- | --- | --- | --- |
| Site | Year |  | Night of survey | Valid tracking plates | Rats | Mice | Shrews |  | Night of survey | Valid tracking plates | Rats | Mice | Shrews |
|  | |  |  |  |  |  |  |  |  |  |  |  |  |
| Askival | |  |  |  |  |  |  |  |  |  |  |  |  |
|  |  |  |  |  |  |  |  |  |  |  |  |  |  |
|  | 2010 |  | 1 | 132 | 3 | 2 | 0 |  | 1 | 119 | 0 | 0 | 0 |
|  |  |  | 2 | 130 | 0 | 1 | 0 |  | 2 | 130 | 2 | 4 | 0 |
|  |  |  | 3 | 132 | 1 | 6 | 0 |  | 3 | 131 | 3 | 8 | 1 |
|  | 2011 |  | 1 | 124 | 1 | 6 | 1 |  | 1 | 117 | 0 | 6 | 4 |
|  |  |  | 2 | 114 | 0 | 3 | 0 |  | 2 | 120 | 1 | 3 | 7 |
|  |  |  | 3 | 127 | 2 | 3 | 0 |  |  |  |  |  |  |
|  | 2012 |  | 1 | 132 | 0 | 0 | 0 |  | 1 | 132 | 1 | 11 | 3 |
|  |  |  | 2 | 130 | 0 | 1 | 0 |  | 2 | 132 | 0 | 8 | 0 |
|  |  |  | 3 | 132 | 3 | 2 | 1 |  | 3 | 130 | 2 | 8 | 4 |
|  | 2013 |  | 1 | 132 | 0 | 3 | 0 |  | 1 | 132 | 2 | 5 | 0 |
|  |  |  | 2 | 132 | 4 | 0 | 0 |  | 2 | 132 | 1 | 8 | 1 |
|  |  |  | 3 | 131 | 0 | 5 | 0 |  | 3 | 132 | 2 | 8 | 3 |
| Clough's Crag | |  |  |  |  |  |  |  |  |  |  |  |  |
|  |  |  |  |  |  |  |  |  |  |  |  |  |  |
|  | 2011 |  | 1 | 128 | 1 | 7 | 0 |  | 1 | 116 | 0 | 0 | 2 |
|  |  |  | 2 | 127 | 3 | 9 | 1 |  | 2 | 128 | 0 | 5 | 10 |
|  |  |  | 3 | 132 | 1 | 3 | 2 |  | 3 | 129 | 0 | 10 | 12 |
|  | 2012 |  | 1 | 132 | 6 | 2 | 1 |  | 1 | 132 | 1 | 0 | 0 |
|  |  |  | 2 | 132 | 0 | 0 | 1 |  | 2 | 132 | 4 | 1 | 0 |
|  |  |  | 3 | 130 | 4 | 3 | 2 |  | 3 | 132 | 8 | 3 | 0 |
|  | 2013 |  | 1 | 132 | 4 | 1 | 2 |  | 1 | 132 | 19 | 2 | 1 |
|  |  |  | 2 | 129 | 0 | 1 | 0 |  | 2 | 130 | 19 | 1 | 0 |
|  |  |  | 3 | 132 | 4 | 3 | 0 |  | 3 | 130 | 16 | 4 | 5 |
|  | |  |  |  |  |  |  |  |  |  |  |  |  |
| Hallival | |  |  |  |  |  |  |  |  |  |  |  |  |
|  |  |  |  |  |  |  |  |  |  |  |  |  |  |
|  | 2010 |  | 1 | 132 | 0 | 0 | 0 |  | 1 | 132 | 0 | 14 | 0 |
|  |  |  | 2 | 132 | 0 | 2 | 0 |  | 2 | 132 | 3 | 32 | 1 |
|  |  |  | 3 | 132 | 1 | 4 | 0 |  | 3 | 131 | 1 | 15 | 1 |
|  | 2011 |  | 1 | 132 | 0 | 7 | 0 |  | 1 | 132 | 0 | 10 | 0 |
|  |  |  | 2 | 132 | 2 | 5 | 0 |  | 2 | 132 | 0 | 4 | 1 |
|  |  |  | 3 | 128 | 2 | 8 | 0 |  | 3 | 129 | 0 | 7 | 4 |
|  | 2012 |  | 1 | 132 | 2 | 1 | 0 |  | 1 | 132 | 2 | 0 | 0 |
|  |  |  | 2 | 132 | 7 | 2 | 0 |  | 2 | 131 | 1 | 6 | 0 |
|  |  |  | 3 | 132 | 1 | 3 | 3 |  | 3 | 132 | 0 | 5 | 0 |
|  | 2013 |  | 1 | 132 | 1 | 13 | 0 |  | 1 | 131 | 1 | 15 | 3 |
|  |  |  | 2 | 132 | 2 | 5 | 0 |  | 2 | 132 | 4 | 11 | 3 |
|  |  |  | 3 | 132 | 2 | 3 | 1 |  | 3 | 132 | 4 | 19 | 9 |
|  |  |  |  |  |  |  |  |  |  |  |  |  |  |
| Total |  |  |  | 4302 | 57 | 114 | 15 |  |  | 4146 | 97 | 233 | 75 |
|  |  |  |  |  |  |  |  |  |  |  |  |  |  |
|  |  |  |  |  |  |  |  |  |  |  |  |  |  |
