# Supplementary material for: Unexpected involvement of a second rodent species makes impacts of introduced rats more difficult to detect
Source: Sci Rep. 2021 Oct 5;11:19805. doi: 10.1038/s41598-021-98956-z (PMC8492617; doi:10.1038/s41598-021-98956-z)
Supplement: Supplementary file 2 — Supplementary Information 2. [file 41598_2021_98956_MOESM2_ESM.docx]

Table S2. Tests for fixed effects site, year, season (early summer, late summer) on the proportion of tracking plates marked by rats, mice or shrews at sites treated with rodenticides and untreated sites. Data were analysed in Generalized Linear Mixed Models with a binomial distribution and logit link function. Terms were sequentially added to the models.

| Untreated sites |  |  |  |  |
| --- | --- | --- | --- | --- |
|  | Fixed term | Wald statistic | F statistic | F pr. |
| Rats |  |  |  |  |
|  | Site | 15.37 | 7.69 | 0.002 |
|  | Year | 12.78 | 4.26 | 0.012 |
|  | Season | 4.43 | 4.43 | 0.043 |
| Mice |  |  |  |  |
|  | Site | 10.96 | 5.48 | 0.009 |
|  | Year | 21.12 | 7.04 | <0.001 |
|  | Season | 3.12 | 3.12 | 0.087 |
| Shrews |  |  |  |  |
|  | Site | 1.33 | 0.67 | 0.521 |
|  | Year | 13.12 | 4.37 | 0.011 |
|  | Season | 11.34 | 11.34 | 0.002 |
| Treated sites |  |  |  |  |
|  | Fixed term | Wald statistic | F statistic | F pr. |
| Rats |  |  |  |  |
|  | Site | 1.02 | 1.02 | 0.327 |
|  | Year | 0.45 | 0.23 | 0.800 |
|  | Season | 0.00 | 0.00 | 0.999 |
| Mice |  |  |  |  |
|  | Site | 5.23 | 5.23 | 0.034 |
|  | Year | 2.06 | 1.03 | 0.375 |
|  | Season | 16.43 | 16.43 | <0.001 |
| Shrews |  |  |  |  |
|  | Site | 1.88 | 1.88 | 0.188 |
|  | Year | 4.43 | 2.21 | 0.140 |
|  | Season | 13.86 | 13.86 | 0.002 |
